# Multiclass classification of diseased grape leaf identification using deep convolutional neural network(DCNN) classifier

**DOI:** 10.1038/s41598-024-59562-x

**Published:** 2024-04-18

**Authors:** Kerehalli Vinayaka Prasad, Hanumesh Vaidya, Choudhari Rajashekhar, Kumar Swamy Karekal, Renuka Sali, Kottakkaran Sooppy Nisar

**Affiliations:** 1 Department of Studies in Mathematics, Vijayanagara Sri Krishnadevaraya University, Ballari, Karnataka India; 2 Department of Mathematics, Manipal Institute of Technology Bengaluru, Manipal Academy of Higher Education, Manipal, Karnataka India; 3 Department of Studies in Computer Science, Vijayanagara Sri Krishnadevaraya University, Ballari, Karnataka India; 4 Department of Mathematics, College of Science and Humanities in Alkharj, Prince Sattam Bin Abdulaziz University, Alkharj, 11942 Saudi Arabia

**Keywords:** Convolutional neural network, Deep neural network classifier, Visual Geometry Group, Support vector machine, Transfer learning, Computational science, Computer science

## Abstract

The cultivation of grapes encounters various challenges, such as the presence of pests and diseases, which have the potential to considerably diminish agricultural productivity. Plant diseases pose a significant impediment, resulting in diminished agricultural productivity and economic setbacks, thereby affecting the quality of crop yields. Hence, the precise and timely identification of plant diseases holds significant importance. This study employs a Convolutional neural network (CNN) with and without data augmentation, in addition to a DCNN Classifier model based on VGG16, to classify grape leaf diseases. A publicly available dataset is utilized for the purpose of investigating diseases affecting grape leaves. The DCNN Classifier Model successfully utilizes the strengths of the VGG16 model and modifies it by incorporating supplementary layers to enhance its performance and ability to generalize. Systematic evaluation of metrics, such as accuracy and F1-score, is performed. With training and test accuracy rates of 99.18 and 99.06%, respectively, the DCNN Classifier model does a better job than the CNN models used in this investigation. The findings demonstrate that the DCNN Classifier model, utilizing the VGG16 architecture and incorporating three supplementary CNN layers, exhibits superior performance. Also, the fact that the DCNN Classifier model works well as a decision support system for farmers is shown by the fact that it can quickly and accurately identify grape diseases, making it easier to take steps to stop them. The results of this study provide support for the reliability of the DCNN classifier model and its potential utility in the field of agriculture.

## Introduction

The cultivation of grapes holds a noteworthy position in the agricultural sector, making a substantial contribution to the economy and serving as a crucial means of subsistence for numerous farmers across the globe. The presence of pests and diseases frequently impedes the growth and productivity of grape plants. The factors mentioned earlier can cause significant reductions in the quality and quantity of agricultural produce, thereby impacting the economic viability and environmental sustainability of grape farming. Plant diseases are a significant concern for grape growers, representing one of their primary challenges. Plant diseases negatively impact grape production, leading to reduced yields and compromised quality that renders them unsuitable for both consumption and commercial purposes. Furthermore, the precise identification and efficient management of these ailments necessitate prompt and precise diagnosis to execute efficacious interventions and curtail damages. In the past, knowledgeable agronomical experts had to conduct a visual examination to identify grape leaf ailments. Nonetheless, this methodology frequently involves subjectivity and consumes time, resulting in delays in executing suitable interventions. Hence, the imperative for the creation of automated and efficient disease diagnosis systems arises, as they are essential in aiding farmers in making timely and precise determinations.

In recent years, rising consumer demand and higher living standards have driven the expansion of grape cultivation. Grapes are a widely consumed fruit that is recognized for its nutritional significance, owing to the presence of diverse advantageous constituents. The active constituents found in grape extracts exhibit antioxidant, antibacterial, anti-inflammatory, and anti-carcinogenic properties, rendering them a valuable resource in the pharmaceutical industry, particularly for the treatment of hypertension. Gavhale et al. ^1^ acknowledged the significance of identifying plant leaf diseases in a facile and uncomplicated manner to facilitate the progress of agriculture. The authors investigated the processes of image acquisition, preprocessing methods for feature extraction, and classification using neural networks. They also analyzed the benefits and drawbacks associated with each of these techniques. According to Ampatazids et al. ^2^, grape cultivation encounters several obstacles during growth, such as vulnerability to unfavorable weather patterns, ecological elements, insect infestations, bacteria, and fungi. Various grapevine foliar diseases, including black rot, black measles, leaf blight, and downy mildew, can have detrimental effects on grape production and quality, resulting in considerable economic losses for grape growers. Zhang et al. ^3^ employed advanced deep learning models, namely enhanced GoogleNet and Cifar10, to achieve accurate identification of leaf diseases. By adjusting the parameters, two enhanced models were devised to facilitate the training and testing of neural networks on a set of nine distinct categories of maize leaf images. Rathnakumar et al. ^4^ presented a framework that provides a rapid, accurate, and pragmatic method for detecting and managing leaf diseases. The methodology employed by the researchers entailed the utilization of a multiclass Support Vector Machine (SVM) grouping algorithm to facilitate the identification and classification of leaf diseases.

In contemporary times, there have been notable developments in deep learning, specifically Convolutional neural networks (CNNs), which have demonstrated encouraging outcomes in diverse image classification assignments. Convolutional neural networks (CNNs) possess the capacity to acquire and extract significant features from images, thereby facilitating the discrimination of distinct object categories. In the context of our study, CNNs are employed to differentiate between various types of grape leaf diseases. Furthermore, the utilization of data augmentation techniques can augment the efficacy of Convolutional neural networks (CNNs) by amplifying the heterogeneity and volume of the training data, thereby resulting in better generalization abilities^5,6^. Numerous investigations have been carried out to examine the identification and assessment of plant ailments through the utilization of deep learning and machine learning methodologies. Ferentinos ^7^ suggested applying Convolutional neural network (CNN) models to identify and classify plant diseases. The methodology involved utilizing basic leaf images of healthy and diseased plants. Barbedo ^8^ proposed a data augmentation technique to enhance the database's variety of images. The approach concentrates on particular lesions and spots rather than the entire leaf. This methodology has improved the ability to identify distinct illnesses impacting a single leaf. Within the realm of grape leaf illnesses, Miaomiao et al. ^9^ established a unified framework using Convolutional neural network s (CNNs) to distinguish between grape leaves affected by common diseases including black rot, esca, and isariopsis leaf spot and unaffected leaves. Using improved Convolutional neural networks (CNNs), Liu et al. ^10^ presented a novel method for disease identification in grape leaves. Their strategy entailed combining photographs from the field with those from open sources. With the use of Convolutional neural networks (CNNs), Hasan et al. ^11,12^ attempted to make it easier to identify and classify diseases in grape leaves. K-means clustering for segmentation, VGG16 transfer learning for feature extraction, and CNNs for classification were only some of the image processing methods used in the authors' research. Mohammed et al. ^13^ developed a method based on artificial intelligence to identify and categorize illnesses affecting grape leaves. In order to accurately diagnose diseases in grape leaves, Lu et al. ^14^ used transformer and ghost-Convolutional networks. Ansari et al. ^15^ used a unique method including support vector machines and image processing to identify and classify grape leaf diseases. Researchers used a multi-stage process that included gathering data, cleaning it up with filters, segmenting with fuzzy C means, extracting features with principal component analysis, and classifying with PSO SVM, BPNN, and random forest. Suo et al. ^16^ created the GSSL method for diagnosing and categorizing illnesses. Grape leaf photos have their texture improved using a variety of image processing techniques, such as the Gaussian filter, Sobel smoothing, denoising, and Laplace operator. For the goal of diagnosing plant illnesses using images of its leaves, Thakur et al. ^17^ proposed using VGG-ICNN, a lightweight Convolutional neural network. In order to speed up the identification of grape leaf diseases, Ashokkumar et al. ^18^ used a region-based Convolutional neural network (CNN) technique, more precisely the Grape Leaf Disease Detection Technique (GLDDT), with a Faster Region based Convolutional neural network (FRCNN). To diagnose illnesses in tomato, apple, and grape plants in real time, Yag et al. ^19^ developed a robust hybrid classification model that combines machine learning and deep learning techniques. The model also includes a swarm optimization-based feature selection procedure. Jeong et al. ^20^ examined two DNN models for the classification and segmentation of plant diseases. By integrating a multiclass support vector machine (SVM) with image processing, Javidan et al. ^21^ provided a different approach to disease detection and classification in grape leaves. Alajas et al. ^22^ go into detail about how to tell normal grape leaves from fungus-infected ones and how to count the total number of spots. This was achieved with the use of cutting-edge tools in computer vision, machine learning, and computational intelligence. To diagnose the mildew disease in pearl millet, Coulibaly et al. ^23^ developed a transfer learning strategy with feature extraction. Results from the pre-trained VGG16 model, which was trained on limited data, were encouraging. For the automatic detection and diagnosis of illnesses affecting rice, maize, and other crops, Chen et al. ^24^ employed the VGGNet and Inception modules. The suggested method significantly outperforms other cutting-edge approaches in terms of performance; on the open dataset, it obtains a validation accuracy of at least 91.83%. The proposed method's average accuracy for classifying photos of rice plants reaches 92.00% even against complicated backdrop conditions. Abbas et al. ^25^ proposed a densenet121 model for tomato disease diagnosis that uses both fake and real photos as training data. Here, the Conditional Generative Adversarial Network (C-GAN) artificially creates images of tomato plant leaves to complement the data. The suggested strategy demonstrates its superiority to the current approaches. Artificial Neural Network (ANN) is a nonlinear statistical model that shows an intricate connection between inputs and outputs in an effort to find a novel pattern. According to the findings of the authors ^26–28^, ANNs are currently the most often used machine learning models that have a wide range of applications in the fields of agriculture, healthcare, environment, finance, etc. to help with decision-making, estimation, classification, and prediction tasks.

This paper describes research that also employs a Deep Convolutional neural network (DCNN) Classifier Model, a type of ANN, for detecting diseases in grape leaves. The primary goal of this study is to classify the many illnesses that might affect grape leaves, such as Black Rot, ESCA, Leaf Blight, and Healthy Leaves. Utilizing the VGG16 architecture with three additional CNN layers achieved the best performance, which in turn improved the generalizability and prediction accuracy of the DCNN classifier. It is vital that farmers immediately and precisely evaluate any issues with the leaves of grape plants in order to reduce the negative consequences of diseases on grape cultivation and maintain the general health and productivity of grape flora.

## Data preparation

### Dataset

**Figure 1:**
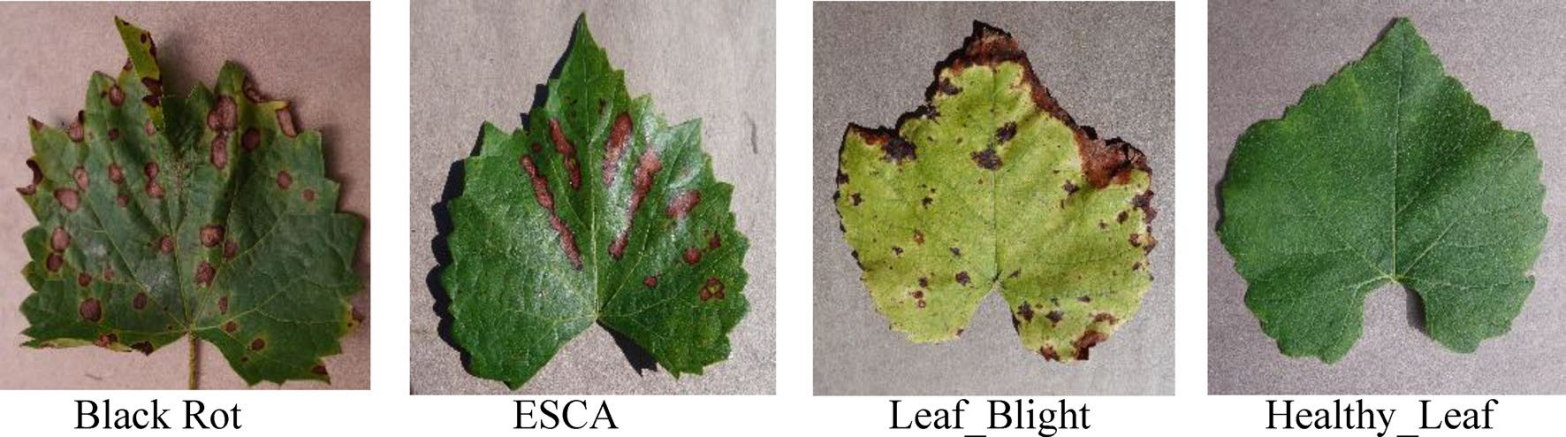
Images of several classes, including those of diseased objects like Black Rot, ESCA, Leaf Blight, and Healthy.

The present investigation sourced its dataset from Kaggle ^29^, a publicly accessible online platform. The grape images in the dataset are all of 256 × 256 pixels in size. As shown in Table 1, the collection is unbalanced and has a total of 9027 photos from four different classes of grape leaf disease. The dataset comprises both asymptomatic and symptomatic images, encompassing manifestations such as black rot, ESCA, and leaf blight symptoms, as presented in Fig. 1. In diagnosing grape diseases, the leaf is utilised instead of the flower, fruit, or stem. The persistent presence of grape leaves, in contrast to the ephemeral appearance of blossoms and fruit, accounts for this phenomenon. Furthermore, the leaf exhibits a higher degree of sensitivity towards the overall health of plants and generally imparts more comprehensive information in contrast to the stem. The grape's stem may not promptly exhibit symptoms of illness.

**Table 1:** Sample grape leaf dataset.

Sl. no	Category	Number of images	Leaf symptoms
1	Black Rot	2360	Appear Small brown circular lesions
2	ESCA	2400	Appear dark red or yellow stripes
3	Leaf Blight	2152	Appear many small rounded, polygonal
4	Healthy	2115	–
Total samples		9027	–

### Data augmentation

**Figure 2:**
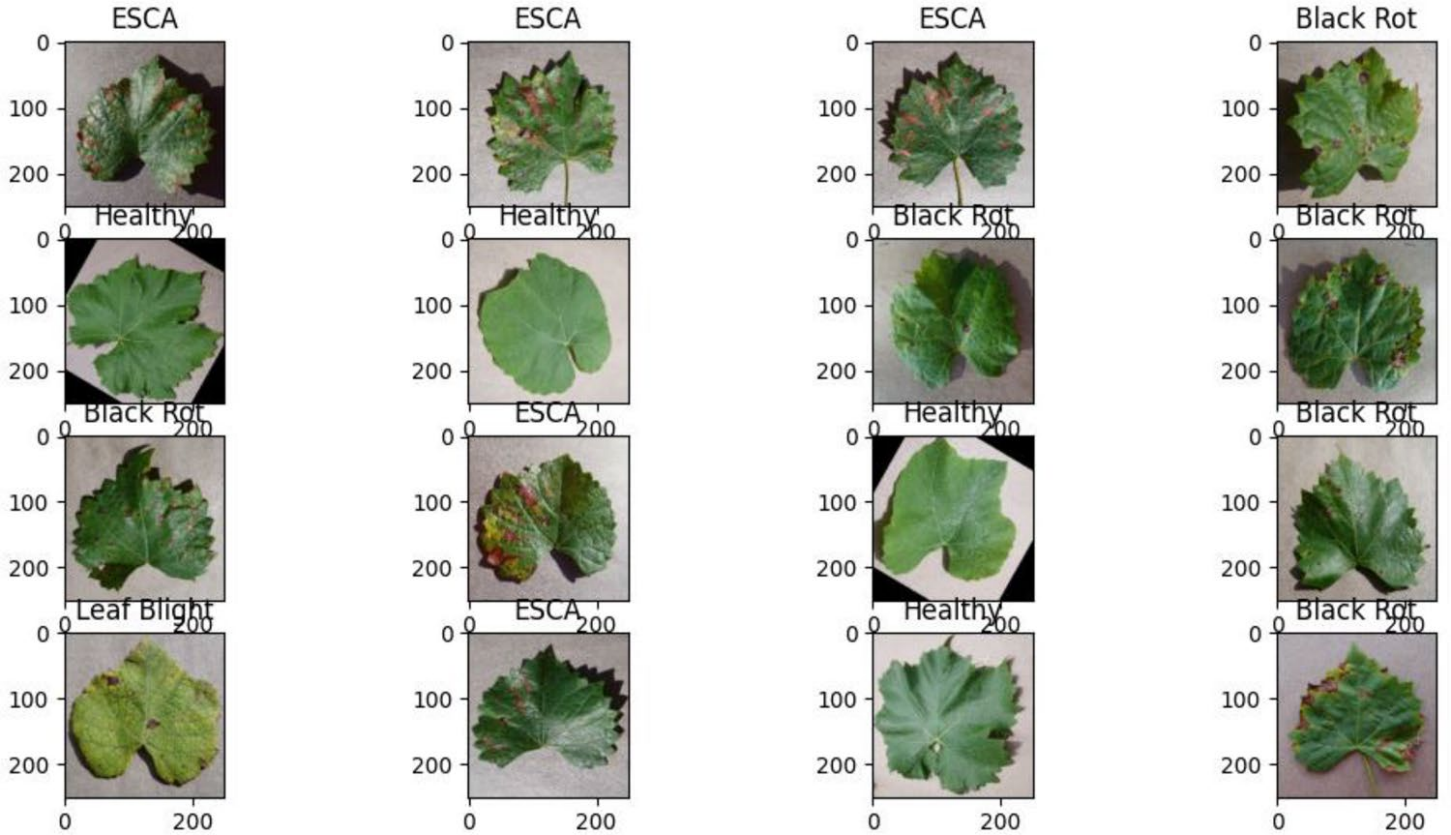
Images after augmentation.

Data augmentation involves applying transformations to existing images to increase the diversity and quantity of training data. Random rotations, flips, zooms, and shifts are commonly used to simulate variations in disease patterns, lighting conditions, and camera angles. Data augmentation improves the model's generalization ability, reduces overfitting risks, and enhances the robustness and accuracy of grape leaf disease image classification (see Fig. 2).

The following transformations are applied to grape leaf images to create augmented images:

- Zoom: Image is resized on the interval [1−x,1 + x]. Here the value of x is 0.1
- Horizontal flip: All the rows and columns of an image pixels are reversed horizontally. Further it creates mirror image of an original image along a vertical line.

### Splitting the data

A total of 7222 images, or eighty percent of the data, were set aside for training, while 1805 images, or twenty percent, were set aside for testing. A further 20% of the training data, i.e., 1444 images were used for validation. The "flow_from_directory()" method of ImageDataGenerator is utilized in the given code to partition the data into training and testing sets while preserving the distribution of classes. The training set has been configured to utilize a portion of the available data by setting the "subset" option to "training". In the context of the testing set, it is observed that the absence of a subset parameter implies the utilization of the entire dataset.

## Methodology

In this study, Convolutional neural network (CNN) models with and without augmentation are developed and compared for their performance. Several techniques, including image preprocessing and data augmentation techniques, are implemented to enhance the classification model's performance. We also further designed a Deep Convolutional neural network (DCNN) classifier model employing the VGG16 architecture with three extra CNN layers and assessed its performance. Finding the model that performed the best for the grape leaf disease classification task was the goal.

### Convolution neural network

At the outset, certain machine learning methodologies commence by extracting features, which are subsequently employed to train a classifier with the aim of automating the classification of leaf diseases. Nevertheless, the process of manually engineering features is a time-consuming endeavor. The rapid progress in deep learning-based models has facilitated researchers efforts to achieve autonomous representation and feature learning ^30^. This study presents the development of a novel convolutional neural network (CNN) architecture that demonstrates the ability to automatically extract features through convolution and pooling. The feature vectors that have been extracted are then utilized for the purpose of categorization.

**Figure 3:**
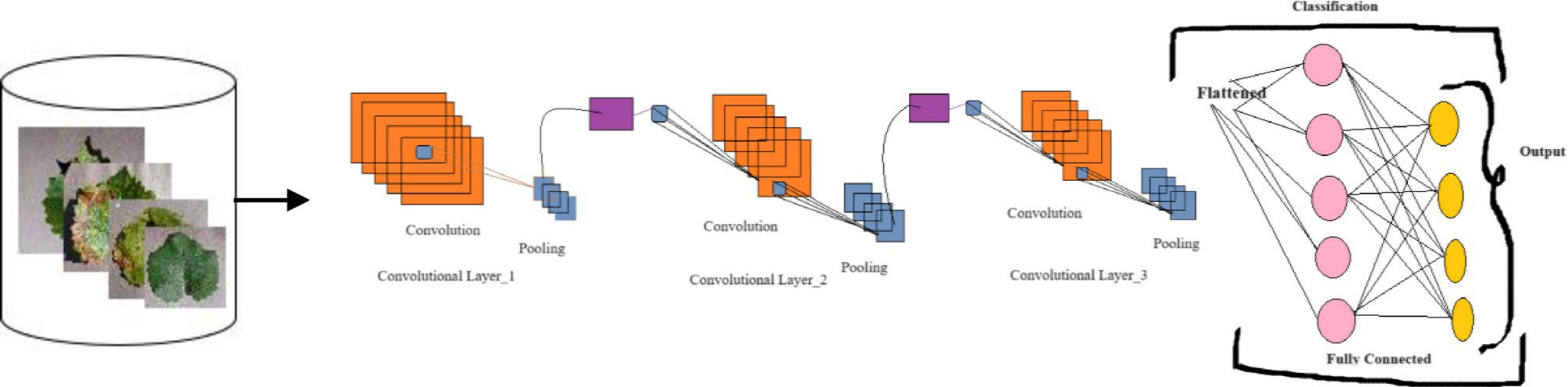
The Block diagram of CNN model’s general framework.

A Convolutional neural network (CNN) is a specialized deep learning algorithm that has been specifically developed for the purpose of analyzing visual data, with a particular focus on images. The advent of computer vision has brought about a significant transformation, resulting in notable achievements in various tasks such as image classification, object detection, and image segmentation. Several published studies have employed Convolutional neural network (CNN) models for the purpose of object recognition and classification ^31,32^. The general framework employed in this study is depicted in Fig. 3. The following is a comprehensive elucidation of each stratum within the proposed conceptual framework.

Convolutional layers are the essential components of a Convolutional neural network (CNN). Filters (or kernels) form these layers and move across the input picture. Feature maps are generated by performing element-wise multiplications and summations. Each filter is optimized for identifying a particular class of visual characteristics, such as edges, corners, or textures. The CNN model incorporates three convolutional layers that utilize 3 × 3 filters.

Pooling layers are commonly used after convolutional layers to decrease the spatial dimensions of the feature maps. A frequently employed pooling technique in the field is known as maxpooling. This technique involves downsampling the feature maps by selecting the maximum value within each pooling region. This procedure facilitates the extraction of essential characteristics while simultaneously mitigating computational complexity. This study employs three maxpooling layers.

The role of activation functions in the architecture of Convolutional neural networks (CNNs) is to introduce non-linearities. Non-linear activation functions, such as the Rectified Linear Unit (ReLU), are frequently employed after each convolutional and fully connected layer. The Rectified Linear Unit (ReLU) activation function is designed to transform the input values in a neural network. It operates by replacing all negative input values with zero while leaving positive input values unchanged. This characteristic of ReLU allows the neural network to effectively learn intricate non-linear relationships between the input and output variables. The utilization of softmax activation is implemented in the ultimate layer to classify four distinct diseases affecting grape leaves.

Fully connected layers are commonly located at the terminal stage of convolutional neural network (CNN) architectures. These layers establish connections between each neuron in a given layer and every neuron in the subsequent layer. This connectivity allows the network to capture and comprehend high-level features, facilitating the generation of predictions. In the context of multiclass classification, it is common to employ a softmax activation function in the output layer. This function facilitates the generation of a probability distribution across various classes.

The loss function serves to measure the discrepancy between the predicted labels and the actual labels. The cross-entropy loss function is frequently employed in classification tasks, often in conjunction with the softmax activation function. The primary goal of training is to minimize the loss function by iteratively adjusting the weights and biases of the network using the backpropagation algorithm. The utilization of a sparse categorical cross-entropy loss function is implemented in this study.

The backpropagation algorithm is utilized to calculate the gradients of the loss function in relation to the parameters of the neural network. Then, optimization algorithms like stochastic gradient descent (SGD) or its variations are used to change the weights and biases in the network based on these gradients. The Adam optimizer is employed in this research. During the training phase, the model's predictions are iteratively improved through the use of backpropagation.

The initial model employed in this study was a Convolutional neural network (CNN) model, which did not incorporate any form of data augmentation. The model was trained using a dataset consisting of labeled photographs. The model's performance was evaluated using an additional validation dataset, and the training accuracy and loss were subsequently recorded. Despite the model's elevated training accuracy, a notable disparity between the training and validation accuracy was observed, thereby indicating the potential occurrence of overfitting.

In order to mitigate overfitting and enhance generalization, a convolutional neural network (CNN) model with augmentation techniques was employed. The training dataset underwent two picture augmentation techniques, namely zoom and horizontal flip. The inclusion of supplementary data expanded the scope and diversity of the training dataset, thereby augmenting the model's capacity for generalization. The monitoring and comparison of training and validation accuracies were conducted to evaluate the influence of augmentation.Algorithm 1 provides a comprehensive description of the training procedure for Convolutional neural networks (CNNs).

**Algorithm 1:**
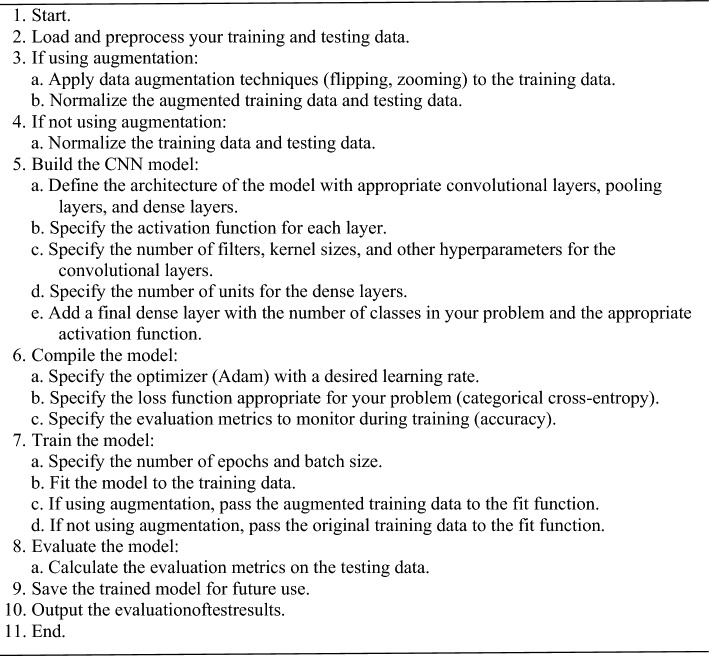
The proposed CNN model.

### Transfer learning

Deep learning algorithms present considerable obstacles, as they necessitate extensive datasets and prolonged training periods due to the multitude of weights and millions of parameters found within deep networks. Data augmentation is a methodology employed to increase the size of a dataset by implementing transformations on images, thus reducing the occurrence of overfitting. Moreover, the utilization of Graphical Processing Units (GPUs) enables the effective allocation of computational resources for the purpose of training deep neural networks.

**Figure 4:**
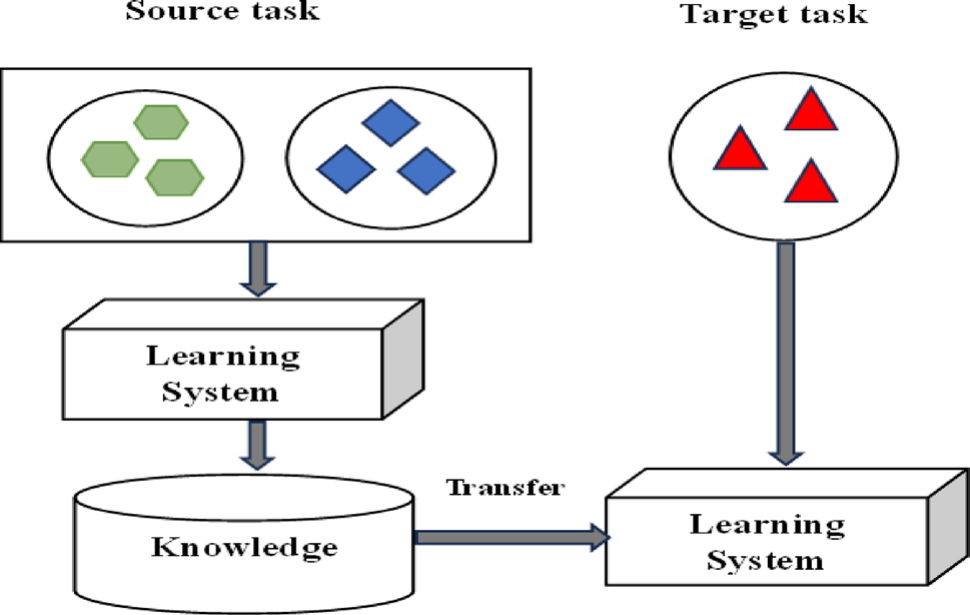
Transfer Learning.

The process of integrating these components requires significant effort and financial resources. Transfer learning has emerged as a viable approach to achieving precise classification with a reduced number of training samples. The concept of transfer learning entails the utilization of a pre-existing model that was initially created for a specific task, but is subsequently employed as a foundation for a distinct yet interconnected task. This practice leads to enhanced performance, as visually represented in Fig. 4.

The goal of this approach is to enhance one's capacity to apply information and abilities learned from one work to another. Convolutional neural network (CNN) designs can be pre-trained, allowing researchers to use features derived from the final layer.These features can be combined with different classifiers prior to the implementation of fully connected layers. The pre-training of these architectures has been shown to result in enhanced performance ^33^. Pan et al. ^34^ provide a comprehensive analysis in which they elucidate the concept of transfer learning and illustrate its practical application in leveraging pre-existing features acquired from one dataset to facilitate training on another dataset.

The existing body of literature presents a wide range of Convolutional neural network (CNN) architectures, which are carefully chosen for specific tasks based on various considerations such as classification accuracy, model complexity, and computational efficiency. The model employed in the study undergoes a series of trials, from which the most successful and efficient model is selected.

## VGG16

The VGG16 architecture, a Convolutional neural network (CNN) proposed by ^35^, is widely acknowledged as one of the most effective vision models currently in existence. The nomenclature "VGG16" is derived from its architecture, which consists of 16 learnable parameters layers, each of which possesses associated weights. The aforementioned robust model accepts a 224 × 224 pixel image as its input and generates a vector of dimensions 1000, which signifies the probabilities associated with each class. The VGG16 architecture consists of a total of 13 convolutional layers, 3 fully connected layers, and 5 pooling layers which sum up to 21 layers but it has only sixteen weight layers. The pooling layers are implemented by employing 2 × 2 filters with a stride of 2, resulting in a reduction of spatial dimensions. In contrast, the convolutional layers employ 3 × 3 filters with a stride of 1 and consistently apply identical padding.

There are three fully connected layers (FC) that terminate the network. Within the realm of architecture, the rectified linear unit (ReLU) is employed as the activation function for each hidden layer. On the other hand, the final fully connected layer uses the softmax activation function to make it easier to classify multiple classes, giving each class a probability. This network is pretty big, with about 138 million parameters. This gives it the ability to learn complex representations and do great work on a wide range of visual tasks.

### Proposed deep convolutional neural network classifier model based on VGG16

**Figure 5:**
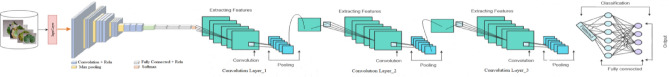
The Block diagram of proposed deep convolutional neural network(DCNN)classifier model’s general framework.

Figure 5 depicts the system implementation of the deep convolutional neural network (DCNN) classifier. The Convolutional neural network (CNN) is trained utilizing the ImageNet datasets, which encompass a vast array of generic images. However, the dataset lacks a substantial number of images specifically pertaining to grape leaf diseases. As a result, the utilization of a pre-existing network would prove insufficient for accurately detecting and classifying diseases affecting grape leaves. Therefore, it becomes imperative to modify and adapt the existing network to effectively address this specific task.

In order to tackle this challenge, we present a proposed model for a deep convolutional neural network (DCNN) classifier that is based on features and designed to promote consistent adaptability. The proposed model leverages a deep learning architecture, wherein a pre-trained model such as VGG16 serves as the fundamental basis. Using the proposed deep convolutional neural network (DCNN) classifier model, the initial 1000 classes in ImageNet can be changed into a more accurate classification of four different diseases that affect grape leaves (as shown in Fig. 5).

Algorithm 2 gives a detailed explanation of how the proposed DCNN classifier model is trained, showing the steps that must be taken in order for accurate disease detection on grape leaves.

**Algorithm 2:**
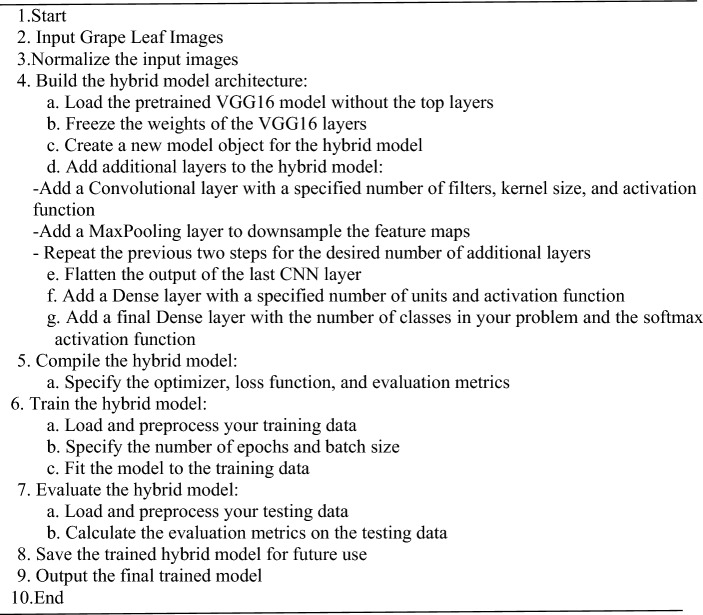
The proposed deep convolutional neural network (DCNN)classifier model.

## Experimental results

### System specification

An Intel Core i5 processor running Windows 11 with 8 GB of RAM was the system used to implement the code. Anaconda, an integrated Python distribution that streamlines deployment and package management, enabled the code execution. Python served as the primary programming language for writing the code, leveraging its versatility and extensive libraries for ML tasks.

### Performance evaluation

The present investigation focuses on the issue of disease identification in grape leaves, specifically as a multiclass classification problem. In this study, a deep convolutional neural network (DCNN) classifier model is employed, which is based on the VGG16 pretrained model. The primary objective of this model is to accurately classify images into different disease categories. To evaluate its performance, the DCNN model is compared with a conventional CNN model. Various techniques are utilized to improve the performance of the classification model, such as data augmentation or non-augmentation, preprocessing, and adjusting the number of epochs.

**Figure 6:**
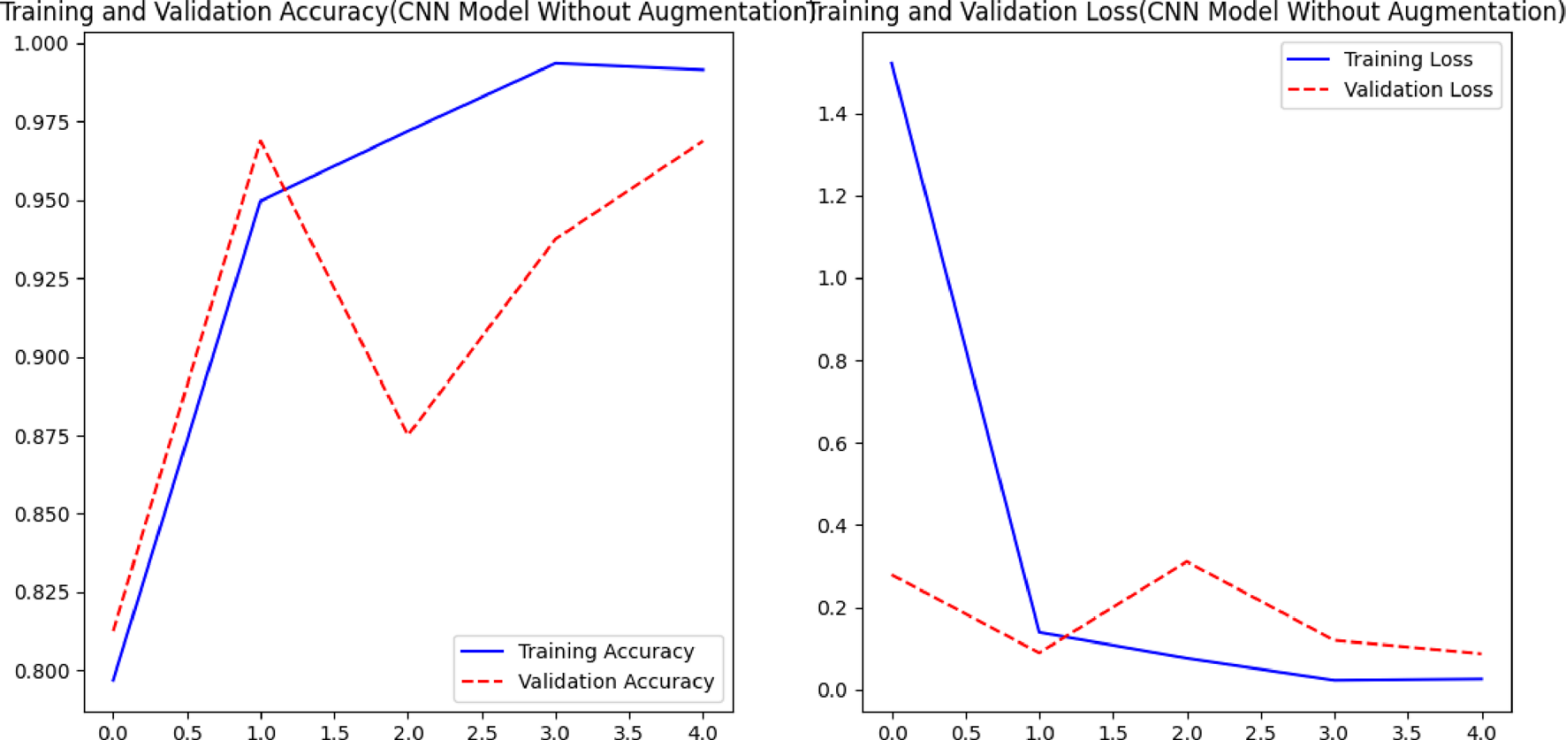
Comparison of Accuracy and loss of CNN Model without Augmentation.

In contrast to previous methodologies, such as those presented by Miaomiao et al. ^9^, our proposed methodology exhibits superior performance. The CNN model, as shown in Fig. 6, shows signs of overfitting because the training accuracy is higher than the validation accuracy. This discrepancy can be attributed to the limited diversity present in the training data, resulting in constrained generalization capabilities.

**Figure 7:**
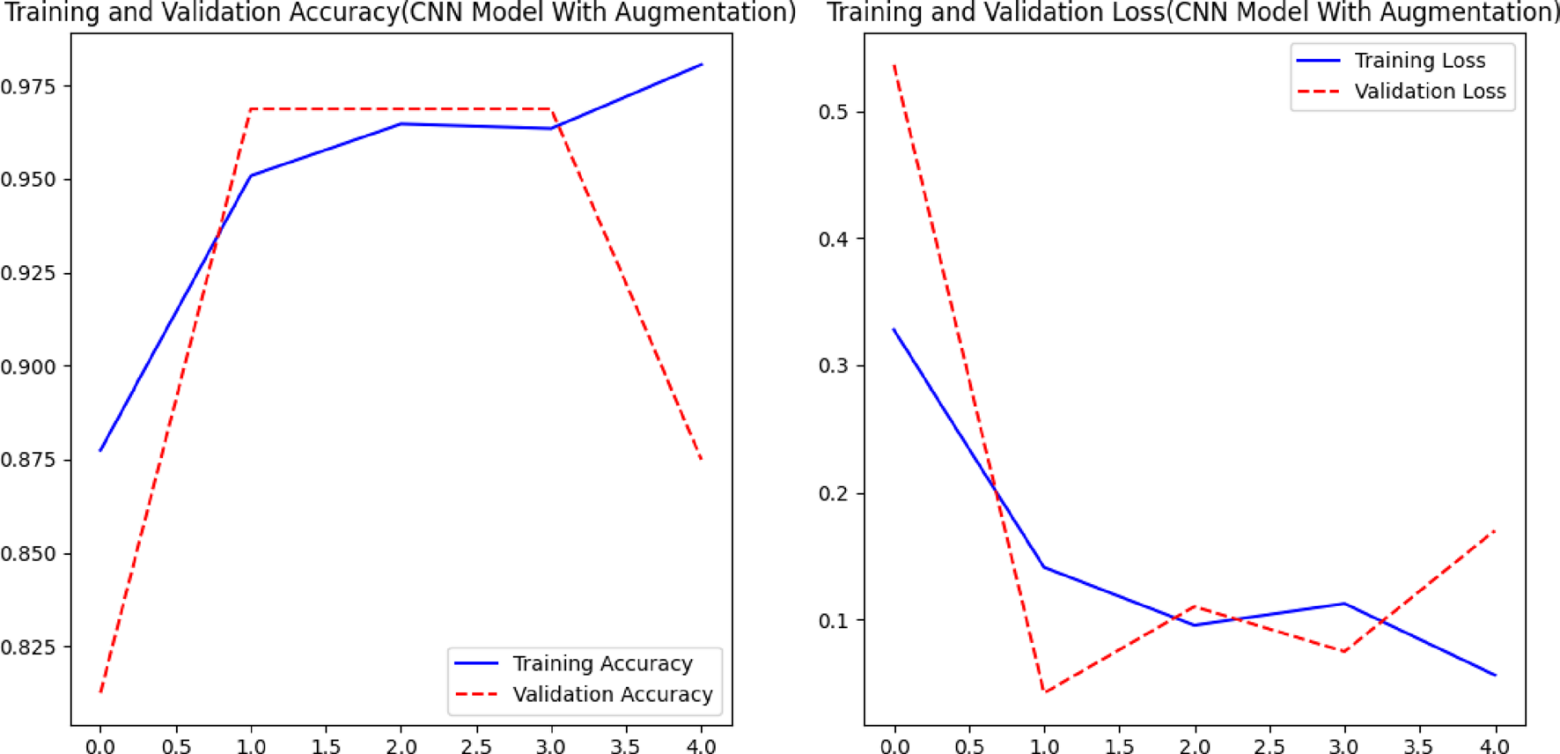
Comparison of Accuracy, Loss of CNN Model withAugmentation.

Using augmentation techniques is one of the most important ways to prevent overfitting and improve the generalizability of models, which improves the accuracy of both training and validation results (see Fig. 6). The accuracy of the CNN model exhibits a consistent upward trend as the number of epochs increases, whereas the loss curve demonstrates rapid convergence (see Fig. 7).

**Figure 8:**
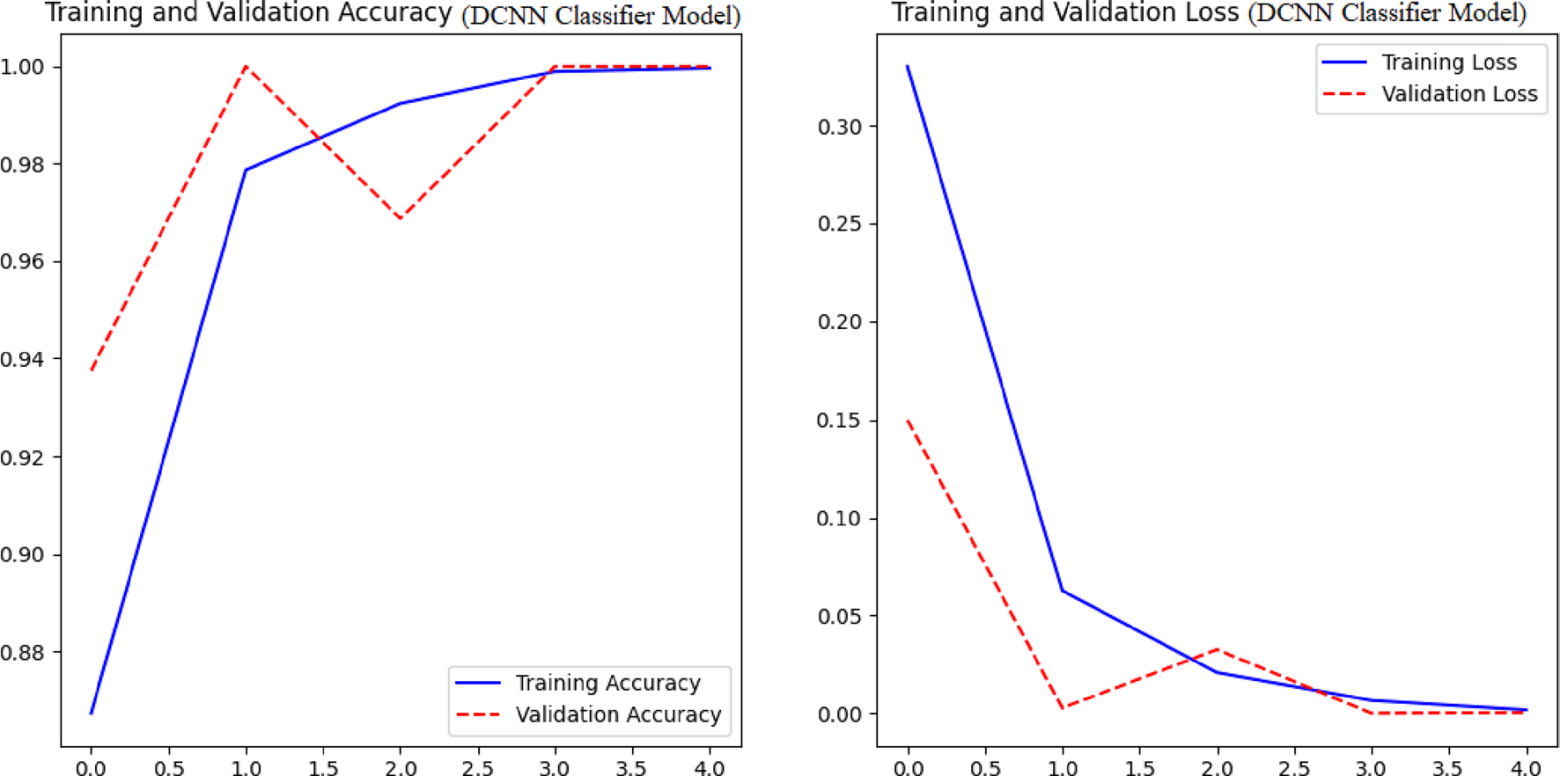
Training performance of DCNN Classifier Model.

**Figure 9:**
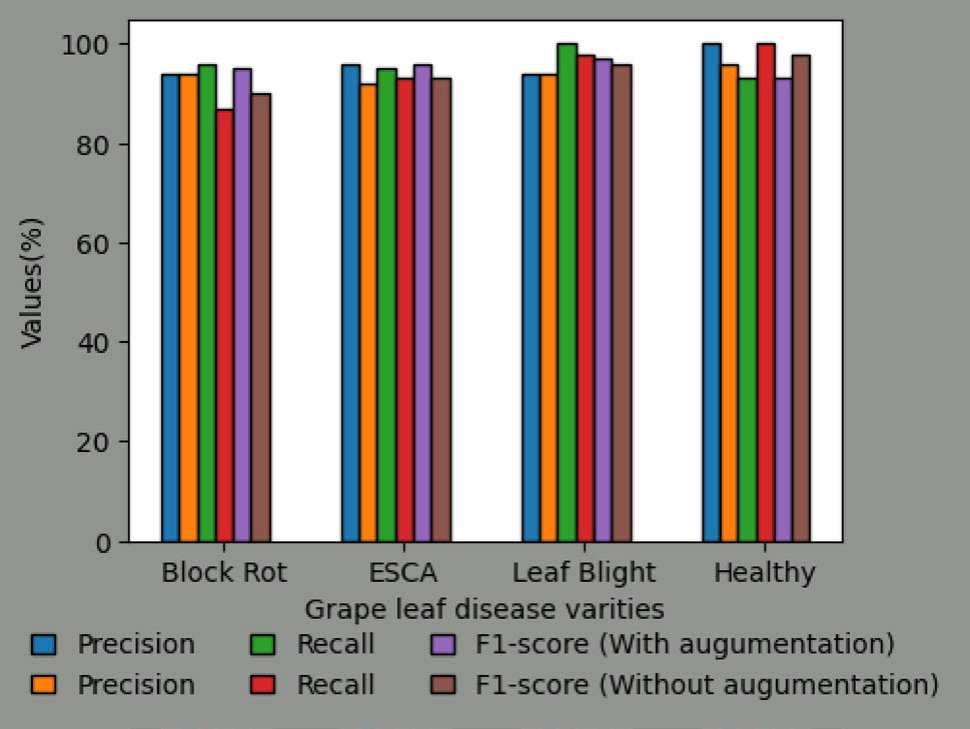
Performance metrics for individual grape leaf disease varities using CNN model with augumentation and without augumentation.

**Figure 10:**
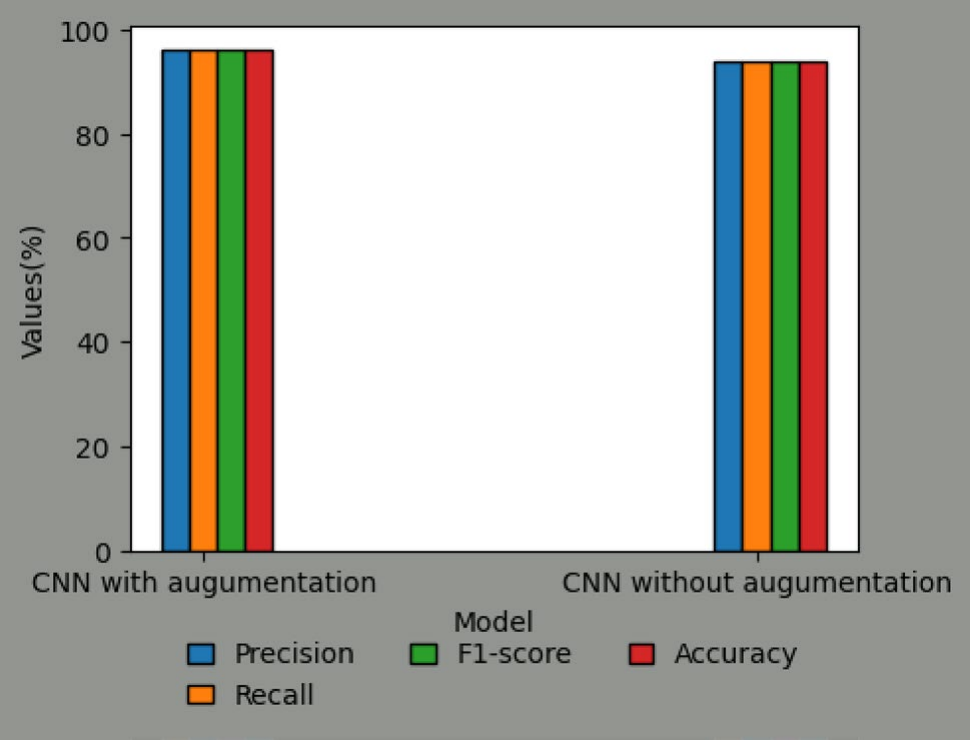
Comparision of CNN model with augumentation and without augumentation.

**Figure 11:**
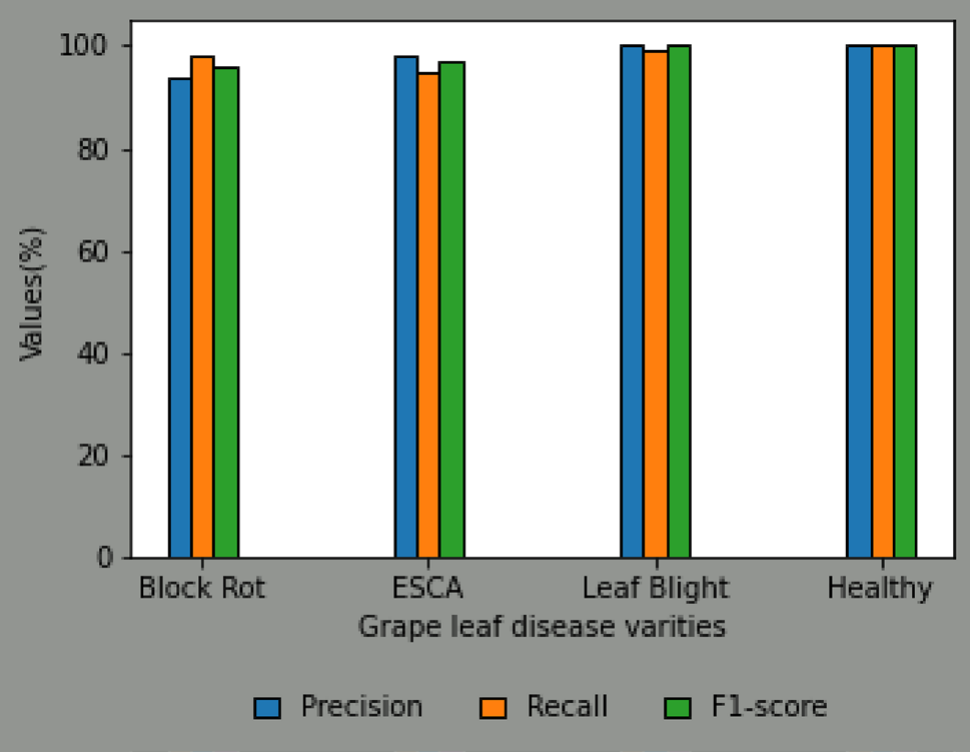
Performance metrics for individual grape leaf disease varities using DCNN Classifier Model.

In the context of the DCNN classification model (as depicted in Fig. 8), it is observed that the training accuracy and loss exhibit a positive trend over the course of training. However, in the absence of augmentation techniques, the model encounters difficulties in effectively generalizing to novel data, resulting in a decline in validation accuracy. The performance metrics for each convolutional neural network (CNN) model on different varieties of grape leaf diseases are illustrated in Fig. 9. Through the process of augmentation, it has been observed that the performance metric values of all disease varieties consistently surpass the threshold of 90%, with the exception of Block Rot. The CNN model with augmentation demonstrates precise categorization accuracy of 100% for the Healthy leaf variety. The CNN model augmented with additional data exhibits superior performance in terms of F1-score, Recall, Precision, and Accuracy, achieving a remarkable 96% (as shown in Fig. 10) compared to the CNN model without augmentation. Figure 11 displays the performance indicators of the DCNN classifier model for each variety of grape leaf disease. With the exception of Block Rot, all disease types exhibit performance measure values exceeding 95%. The DCNN classifier model demonstrates a precision rate of 100% in accurately classifying the Healthy and Leaf Blight varieties.

Based on the data presented in Table 2, it is apparent that the DCNN classifier model exhibits superior performance in terms of F1-score, Recall, Precision, and Accuracy. Specifically, the DCNN model achieves a remarkable 99% in these metrics, surpassing the CNN model utilized in this investigation by a margin of 3%. The DCNN Classifier Model, which was implemented using the open-source Keras framework built on TensorFlow, demonstrates a notable precision of 99% when evaluated on the test data. This surpasses the previously reported results have shown in the given below Table 2.

**Table 2:** DCNN Model performance on the test data.

Leaf type	Precision	Recall	F1-Score
Black Rot	0.99	0.98	0.98
ESCA	0.99	0.99	0.99
Healthy	1.00	1.00	1.00
Leaf Blight	0.99	1.00	0.99
Accuracy	0.99		
Micro Avg	0.99	0.99	0.99

**Figure 12:**
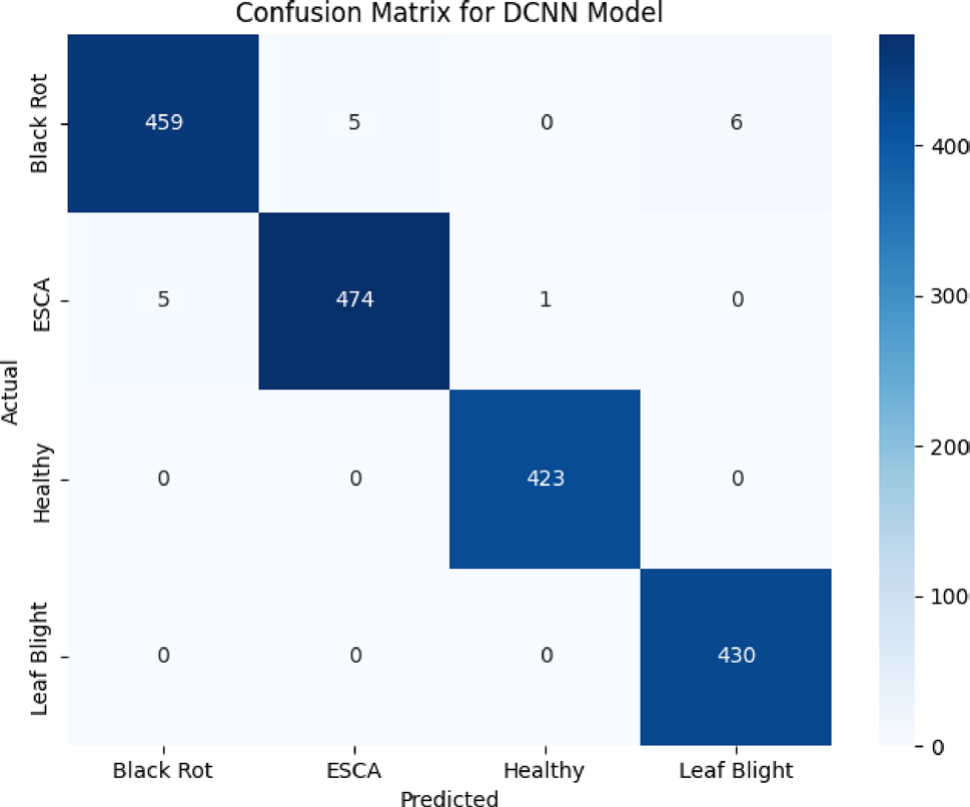
Confusion matrix for DCNN Model.

To assess the performance of the models, a confusion matrix is displayed in Fig. 12. The DCNN model outperformed the other two models through analysis of the confusion matrix, which had 1805 testing images. Whole number representations are given for the confusion matrix.The healthy leaf type is classified perfectly followed by ESCA, Leaf Blight and Black Rot.

### Comparative analysis

The performance comparison between the suggested DCNN model and a number of models from the literature is shown in Table 3. A 98.57% test accuracy was achieved by Miaomiao et al. ^9^ using the UnitedModel architecture. Liu et al. ^10^ obtained a somewhat lower test accuracy of 97.22% using the DICNN architecture. Hasan et al. ^11^ reported a 91.37% test accuracy using a CNN architecture. Out of all the models tested, the Deep Convolutional neural network (DCNN) architecture utilized in this study yielded the best test accuracy, at 99.06%.

**Table 3:** Comparision study.

Authors name	Architecture	Test accuracy (%)
Miaomiao et al. ^9^	UnitedModel	98.57
Liu et al. ^10^	DICNN	97.22
Hasan et al. ^11^	CNN	91.37
Present study	DCNN	99.06

In this study, DCNN model is developed based on the popular and extensively used VGG16 architecture, utilizes the information encoded in the pretrained VGG16 layers. And the additional Convolutional and MaxPooling layers added to the DCNN classifier helps to learn task-specific features and provide the flexibility required to capture complex patterns in the grape leaf disease data. In overall, this optimization technique leads to better performance by combining the benefits of transfer learning with the adaptability to the unique details of the grape leaf disease identification task.

## Conclusion

This article presents a proposed method that is effective in distinguishing between leaves that are healthy and those that are diseased. The research employs Convolutional neural networks (CNN) both with and without augmentation techniques, in addition to a DCNN Classifier model that is based on the VGG16 architecture. Augmentation has been recognized as a valuable technique for improving generalization capabilities and mitigating overfitting issues in convolutional neural network (CNN) models. The Deep Convolutional Neural Network (DCNN) model does better than other models because it uses the VGG16 architecture and adds additional Convolutional Neural Network (CNN) layers. This highlights its aptness for tasks involving image classification. Demonstrating a training data accuracy of 99.18% and a test data accuracy of 99.04%, the deep convolutional neural network (DCNN) classifier shows a lot of promise for accurately detecting grape leaf diseases. In order to improve the performance of grape leaf disease classification, future research could look into new augmentation techniques, optimization of hyperparameters, and integration of state-of-the-art deep learning architectures.

## Data Availability

The datasets generated and/or analysed during the current study are available in the [Rajarshi Mandal] repository, [https://www.kaggle.com/datasets/rm1000/grape-disease-dataset-original].
